# Two severe cases of H7N9 pneumonia patients with immunoneuroendocrine axis dysfunction and vitamin D insufficiency

**DOI:** 10.1186/1471-2334-14-44

**Published:** 2014-01-28

**Authors:** Jin Yao, Linhua Liu, Gang Chen, Leng Lin

**Affiliations:** 1 Department of Infectious Disease, Fujian Provincial Hospital, Fujian Medical University, Fuzhou 350001, China; 2 Department of Endocrinology, Fujian Provincial Hospital Key Laboratory of Endocrinology, Fujian Medical University, Fuzhou 350001, China; 3 Department of Infectious Disease, Fujian Provincial Hospital, Fujian Medical University, Box 4-704, 92 Huqian Road, Fuzhou, Fujian, China

**Keywords:** H7N9 pneumonia, Vitamin D insufficiency, Immunoneuroendocrine axis

## Abstract

**Background:**

The immunoneuroendocrine axis plays a major role in the regulation of the host’s response to infection, but its role in severe H7N9 pneumonia is still unknown. Therefore, this study is carried out to explore the relationship between the immunoneuroendocrine axis and severe H7N9 pneumonia.

**Case presentantion:**

The study included two H7N9 pneumonia patients. Endocrine response and cellular immune function in prolonged phase of these two severe H7N9 pneumonia cases were reported and analyzed. A 57-year-old male patient (case 1) and a 68-year-old male patient (case 2) were admitted because of cough, fever and dyspnea. Moist rales were present in both lungs. The rest of the examination was reportedly normal. The laboratory test showed that (1) The patients had loss of cortisol rhythm and elevated cortisol level at 4 pm. (2) The patients showed decline of cellular immune function. (3) The patients showed vitamin D insufficiency. (4) Case 2 had higher cortisol level but lower DHEAS, serum phosphorus and vitamin D level as well as cellular immune function than case 1. (5) The thyroid axis, gonadal and lactotropic axis were normal, so were the level of FT3, FT4, STSH and LH, FSH, T, E2 as well as PRL in these two cases. Chest CT revealed inflammation of both lungs especially in right lung. Real time RT-PCR by Centers for Disease Control and Prevention (CDC) confirmed H7N9 infection.

**Conclusion:**

Immunoneuroendocrine axis dysfunction may play an important role in severe H7N9 pneumonia. We need pay more attention to hypophosphatemia and vitamin D insufficiency in H7N9 pneumonia.

## Background

Since the first human infection with influenza A (H7N9) viruses has been identified in Shanghai on March 31, 2013, the latest variant of the avian flu virus has spread across ten provinces in China. Clinical data have been collected to study the immunoneuroendocrine axis in prolong phase of severe H7N9 pneumonia since two H7N9 pneumonia patients were admitted to Fujian Provincial Hospital on April 29,2013. To our knowledge, no study had specially addressed the role of immunoneuroendocrine axis in prolong phase of severe H7N9 pneumonia.

## Case presentation

A 57-year-old male patient (case 1) and a 68-year-old male patient (case 2) were admitted because of cough, fever and dyspnea. It is reported that they had a history of contact with poultry. Case 1 had been well before admission and so was Case 2 except for some symptoms of coronary disease and chronic obstructive pulmonary disease (COPD) before admission. On examination, the temperature of case 1 and case 2 were 39°C and 39.7°C respectively, pulse were 85 beats/min and 78 beats/min respectively, respiratory rate were 25 breaths/min and 30 breaths/min respectively, and blood pressure were 143/76 mmHg and 112/78 mmHg respectively. The patients were alert, oriented but appeared fatigued and sick. They were in shortness of breath and with cyanotic lips. Moreover, moist rales were present in both lungs. Chest CT revealed inflammation of both lungs especially in right lung. While, the rest of the examination was reportedly normal. The laboratory test was shown in Table 1. RNA was extracted from throat-swab samples with the RNeasy mini kit (Qiagen, Valencia, CA, USA) as per the manufacturer’s protocol and tested by real-time RT-PCR withH7N9-specific primers and probes as previously described [1]. The specific sequences have been published on the WHO website at http://www.who.int/influenza/gisrs_laboratory/a_h7n9/en/. Real time RT-PCR by CDC confirmed H7N9 infection. The functions of thyroid, adrenal, gonad and cellular immune system, and the levels of PRL, PTH, 25OH-VitD were examined one week after methylprednisolone administration stopped. The immunoneuroendocrine axis test showed that (1) The patients had loss of cortisol rhythm and elevated cortisol level at 4 pm. (2) The patients showed decline of cellular immune function. (3) The patients showed vitamin D insufficiency. (4) Case 2 had higher cortisol level but lower DHEAS, serum phosphorus and vitamin D level as well as cellular immune function than case 1. (5) The thyroid axis, gonadal and lactotropic axis were normal, so were the level of FT3, FT4, STSH and LH, FSH, T, E2 as well as PRL in these two cases. Two patients were diagnosed severe H7N9 pneumonia with mild acute respiratory distress syndrome. The patients received antiviral treatment with oseltamivir and cefoperazone was used to protect against infection. They were also administered methylprednisolone 40 mg/d for 3 days. Non-invasive mechanical ventilation, thymalfasin, rocalirol and supportive treatment were provided as well. The patients were discharged from hospital after three weeks.

**Table 1:** Laboratory data of study subjects

**Laoratory data**	**Case 1**	**Case2**	**Normal range**
**Blood cell count**			
WBC (× 109/L)	2.0 × 10^9^	2.7 × 10^9^	4–10
N (%)	68	67	50–75
L (%)	22	23	20–40
**Blood levels of electrolytes**			
Serum sodium (mmol/L)	131	125	135–145
Serum potassium (mmol/L)	4.8	4.4	3.5–5.5
Corrected serum calcium (mmol/L)	2.13	2.27	2.25–2.65
Serum phosphorus (mmol/L)	1.04	0.55	0.80–1.48
Serum magnesium (mmol/L)	0.95	0.85	0.6–1.2
**Renal-function tests**			
Serum uric acid (mmol/L)	163	106	237–357 (male)
Creatinine (umol/L)	67	73	40–135
BUN (mmol/L)	4.1	5.3	2.1–7.1
**Liver-function tests**			
ALT (U/L)	83	16	5–40
AST (U/L)	83	47	5–42
Albumin (g/l)	35	27	35–55
**D dimmer (μg/ml)**	2.4	2.6	<0.5
**Blood gas analysis**			
PH	7.38	7.47	7.35–7.45
PCO_2_ (mmHg)	37.1	27	35–45
PO_2_ (mmHg)	57.7	63	90–100
**Cellular immune function**			
CD3 (%)	48	48	55–84
CD3 (/Ul)	440	273	690–2540
CD4 (%)	30	21	31–60
CD4 (/Ul)	279	120	410–1590
CD8 (%)	16	26	13–41
CD8 (/Ul)	152	148	190–1140
CD4/CD8	1.88	0.81	1.05–2.03
NK (%)	25	30	5–27
NK (/Ul)	256	201	90–590
**Fasting blood glucose (mmol/l)**	6.8	4.8	4.0–5.5
**Thyroid axis**			
FT3 (pmol/l)	4.94	3.37	3.1–6.8
FT4 (pmol/l)	21.15	21.98	12–22
sTSH (mIU/l)	0.85	0.37	0.27–4.2
**Lactotropic axis**			
Prolactin (ng/ml)	8.79	11.13	1.64–13.13
**Gonadal axis**			
LH (IU/l)	8.27	10.96	1.25–8.62
FSH (IU/l)	8.0	21.1	1.27–19.26
T (nmmol/l)	21.2	8.69	6.07–27.24
E2 (pg/ml)	46	21	20–47
**Adrenal axis**			
ACTH 8 am (pg/ml)	27.5	22.2	7.2–63.6
Cortisol 8 am (nmol/l)	364	488	240–680
Cortisol 4 pm (nmol/l)	307.8	406.5	<276
DHEAS (ug/dl)	167.6	45.4	38–313
**Parathyroid hormone** (Pg/ml)	5.3	36.7	15–88
**25OH-VitD** (ng/ml)	29	13.9	30–100
**Procalcitonin** (ng/ml)	0.31	0.26	<0.05
**Erythrocyte sedimentation rate** (mm/h)	17	22	0–15

## Conclusion

In our study, the two cases showed the loss of cortisol rhythm, elevated cortisol level at 4 pm and decline of cellular immune function in prolonged phase of H7N9 pneumonia. Interestingly, case 2 had higher cortisol level and lower DHEAS levels than case 1, but had lower cellular immune function. One meta-analysis also has demonstrated the association between high cortisol levels and mortality, which made cortisol an useful biomarker for assessing prognosis in patients with severe community-acquired pneumonia (CAP) [2]. Glucocorticoids influence the traffic of circulating leukocytes and inhibit many functions of leukocytes and immune accessory cells [3]. They inhibit cell accumulation at inflammatory sites and reduce the number of circulating lymphocytes, monocytes, and eosinophils by inducing cell apoptosis [4]. Conversely, cytokines, produced by activated immune cells and neuroendocrine cells as well, are able to modulate the hypothalamus-pituitary-adrenal (HPA) axis at each level: the hypothalamus, pituitary, and adrenal glands [5]. DHEAS is a pleiotropic adrenal hormone, primarily regulated by corticotropin, with proimmune and proinflammatory effects, opposing the immunosuppressive effects of glucocorticoids. The high glucocorticoid level and low DHEAS level suggest an imbalance between immunosuppressive and immunostimulatory adrenocortical hormones, which can result in increased susceptibility to infectious complications during the chronic phase of severe illness.

The level of vitamin D in the two patients were all under normal range and case 2 showed hypophosphatemia. Although some studies had shown that vitamin D insufficiency and hypophosphatemia might weaken the host’s immune defense [6,7], whether or not low level of serum phosphorus and vitamin D would lead to adverse outcome in H7N9 remained unknown. Therefore further studies should be conducted to answer the aforementioned question. One limitation of this study was that we didn’t know whether our patients had low level of 25OH-VitD and serum phosphorus before they had H7N9 pneumonia. But we did need pay more attention to hypophosphatemia and vitamin D insufficiency in severe H7N9 pneumonia. Thus, we used rocalirol to correct vitamin D deficiency in our patient. In our opinion, vitamin D should be measured in severe H7N9 Pneumonia.

The thyroid axis, gonadal and lactotropic axis were normal, so were the level of FT3, FT4, STSH and LH, FSH, T, E2 as well as PRL in these two cases we studied because they were in prolonged but not acute phase of infection.

In conclusion, our cases report suggested that immunoneuroendocrine axis dysfunction might play an important role in severe H7N9 pneumonia. We need pay more attention to hypophosphatemia and vitamin D insufficiency in H7N9 pneumonia.

## Consent

Written informed consent was obtained from the patient for publication of this case report and any accompanying images. A copy of the written consent is available for review by the Editor of this journal. This study was proved by IRB of Fujian Provincial Hospital.

## Competing interests

The authors declare that they have no competing interests.

## Authors’ contributions

JY participated in analysis, interpretation of data and wrote the manuscript. Li L and GC participated in acquisition of data and design. Le L participated in design, interpretation of data and gave final approval of the version to be published. All authors read and approved the final manuscript.

## Pre-publication history

The pre-publication history for this paper can be accessed here:

http://www.biomedcentral.com/1471-2334/14/44/prepub
